# The interplay between gut bacteria and the yeast *Candida albicans*

**DOI:** 10.1080/19490976.2021.1979877

**Published:** 2021-09-29

**Authors:** J. Christian Pérez

**Affiliations:** Department of Microbiology and Molecular Genetics, McGovern Medical School, the University of Texas Health Science Center at Houston, Houston, USA

**Keywords:** Gut bacteria, mycobiota, *candida albicans*, inter-kingdom interactions, fungi

## Abstract

The fungus *Candida albicans* is a ubiquitous member of the human gut microbiota. Hundreds or thousands of bacterial taxa reside together with this fungus in the intestine, creating a milieu with myriad opportunities for inter-kingdom interactions. Indeed, recent studies examining the broader composition – that is, monitoring not only bacteria but also the often neglected fungal component – of the gut microbiota hint that there are significant interdependencies between fungi and bacteria. Gut bacteria closely associate with *C. albicans* cells in the colon, break down and feed on complex sugars decorating the fungal cell wall, and shape the intestinal microhabitats occupied by the fungus. Peptidoglycan subunits released by bacteria upon antibiotic treatment can promote *C. albicans* dissemination from the intestine, seeding bloodstream infections that often become life-threatening. Elucidating the principles that govern the fungus-bacteria interplay may open the door to novel approaches to prevent *C. albicans* infections originating in the gut.

The intestinal microbiota is a consortium of microorganisms including bacteria, fungi, protists, archaea, and viruses. Although the vast majority of studies in the microbiota field are still focused on the bacterial component, the last decade has seen a steady increase of research featuring the biology and critical importance of non-bacterial members of this microbial community.^1–5^Gut commensal fungi (reviewed in Ref. 5) have become a subject of particular interest because recent studies are revealing beneficial effects of fungal colonization for the mammalian host including resistance to pathogens and tuning of the immune system,^6,7^ as well as roles in human diseases such as inflammatory disorders and specific cancers.^8^ Yet neither fungi nor any other microbial taxa are likely to act alone in such a crowded ecosystem. The fact that fungi cohabit with bacteria in the human intestine rather implies that myriad interactions occur between members of these two kingdoms. In this review, I highlight current developments in the study of fungal–bacterial interactions that take place in the mammalian gut. While research looking at the entire gut fungal community is outlined in some sections of the paper, the focus of the review is on the most prevalent fungus in the human intestinal tract, *Candida albicans*.^9^

## Interdependencies between bacteria and fungi in the mammalian gut

There is a large body of literature examining how particular bacteria modulate fungal traits (*e.g*. in *C. albicans*) and *vice versa*. However, the vast majority of the data on bacteria–fungi interactions have been collected using either *in vitro* systems (*e.g*. biofilm formation) or non-intestinal mucosal settings (*e.g*. oral or vaginal). Much of this literature is neatly summarized in recent, comprehensive reviews^10–13^and will not be detailed here. A fundamental question that should be asked, however, is whether the interactions and effects that have been documented under *in vitro* settings (*e.g*. co-culture under laboratory conditions) reflect in any way what takes place in the intestinal tract. While the paucity of mechanistic studies in well-defined mammalian animal models precludes an answer to this question in many specific cases, broader analyses and observations clearly support the notion that there are interdependencies between fungi and bacteria in the gut. The interdependencies can be seen both ways: Bacteria impinging upon fungi and *vice versa*.

### Eliminating bacteria through antibiotic treatment results in fungal dysbiosis

The yeast *C. albicans* is a dweller of the human gastrointestinal tract (reviewed in Ref. 13) along with hundreds (or even thousands) of bacterial taxa. As early as in the 1960s it was observed that antibiotic treatment in humans resulted in *C. albicans* overgrowth,^14,15^ presumably due to the dampening of competing microbes. More recent studies equipped with the tools to monitor the entire intestinal fungal community have revealed that antibiotic treatments that target bacteria inadvertently alter the fungal composition as well.^16,17^ Thus, fungal dysbiosis can result from eliminating or reducing gut bacteria.

### Changes in gut fungal communities lead to bacterial dysbiosis

Evidence is also accumulating that targeting fungi can lead to changes in bacterial communities. For instance, in one of these studies mice treated with antifungal drugs exhibited pronounced alterations in the composition of their bacterial community.^18^ In another study in gnotobiotic mice, a small community of fungi (five species) was found to induce strong ecological changes in the assembly of gut bacteria.^19^ *Candida albicans* itself has been shown to impact the reassembly of gut bacterial communities after antibiotic treatment.^20,21^ The fungus also has a role in the ability of intestinal microbial consortia to resist change in response to antibiotic disruption, which is termed ecological resistance.^22^ Furthermore, gut fungal dysbiosis has been associated with reduced efficacy in fecal transplants currently in use to treat recurrent *Clostridium difficile* infections.^23^

In a longitudinal study of a cohort of 178 preterm infants in which the assembly of the intestinal microbiota was monitored, Rao et al.^24^ were able to infer several interdependencies between specific fungi and bacterial taxa. For instance, they inferred that *C. albicans* antagonizes *Klebsiella pneumoniae* whereas *Staphylococcus* spp. may antagonize *Candida*. Some of the predicted pairwise interactions were further confirmed in gut colonization experiments in rodents indicating that, at least in microbial communities of relatively low complexity such as the infant gut, it may be possible to single out significant fungus-bacteria interactions. Another large study (942 human adults) found a positive correlation between fungal and bacterial richness in the gut, with is suggestive of mutualism between both kingdoms, yet failed to uncover correlations between fungi and bacteria at the species level.^25^ Taken together, the studies described in this section, while short of providing mechanistic details, do indicate that fungi and bacteria impact on the proliferation of each other in the mammalian gut.

## The yeast *Candida albicans* is a pathobiont of the human intestine

Several yeasts of the genus *Candida* are considered true symbionts of the human gut.^26^ *Candida albicans*, however, is the species of the genus *Candida* most frequently detected in feces of healthy humans.^27–29^ This species, therefore, is considered a ubiquitous member of the human gut microbiota. *Candida albicans* appears to have no major environmental reservoir, suggesting that it has extensively coevolved with humans and cohabiting microbes. The fungus can be found in other body sites beyond the intestine (*e.g*. mouth, skin, vagina) and is a common cause of fastidious mucosal disease in otherwise healthy people.^30^ *C. albicans* can also disseminate from the human gut into the bloodstream and invade internal organs producing invasive, life-threatening infections.^31,32^ Invasive *Candida* infections rank in the top four hospital-associated bloodstream infections in the United States.^33,34^ Common risk factors for invasive candidiasis include intensive care unit stay, central venous catheter use, broad-spectrum antibiotics, recent abdominal surgery and immune suppression.^33,35^

*Candida albicans* cells can adopt a variety of specialized morphologies that considerably differ from one another in gene expression profile, cell surface composition and how they interact with host cells.^36–40^ Two of the most readily found *C. albicans* morphologies are the oval-shaped ‘yeast’ form and the long filaments termed ‘hyphae.’ The yeast and hyphae morphologies are not only easy to score but also the transition between both forms is critical for the fungus to invade host tissues and cause disease (see Ref. 40 for a recent review). The yeast-to-hyphae transition can be driven by numerous environmental signals, including temperature, presence of serum and CO_2_, among others. As described in the following sections, many studies examining whether bacteria impinge upon the biology of *C. albicans* have looked at effects on the yeast-to-hyphae transition.

While less studied in the context of microbial interactions, other *C. albicans* cell morphologies such as white/opaque^41^ and GUT (gastrointestinally induced transition)^42^ may also be relevant for the fungus in the intestine. The *WOR1* transcript, which encodes the “master” regulator of white-opaque switching, was strongly upregulated by *in vitro* co-culture with the intestinal bacteria *K. pneumoniae, Escherichia coli*, and *Enterococcus faecalis*.^43^ In addition to *WOR1*, the authors found a number of opaque-specific genes also upregulated in the co-culture experiment, yet the full opaque-specific gene expression pattern was not observed.^43^ Employing a *C. albicans* strain genetically modified to overproduce *WOR1*, another group reported that in the murine intestine the fungus adopted a morphology resembling – but distinct from – that of opaque cells.^42^ The transition to these so-called GUT cells was postulated to confer enhanced fitness in the mammalian gastrointestinal tract.^42^

## Direct effects of bacterial products on *C. albicans*

Gut bacteria could influence the proliferation of *C. albicans* or other fungi (or impact any other fungal trait) by at least two broad mechanisms: One is the release of molecules such as cell surface components, peptides or metabolic products that can directly impact the biology of the fungus. An alternative scenario involves an indirect route in which bacterial molecules (or the microbes themselves) elicit responses from host cells; in this scenario, the microbe-induced host response is what ultimately targets the fungus. I detail here a few examples illustrating the former mechanism and will cover the latter scenario in the following section.

**Figure 1.**
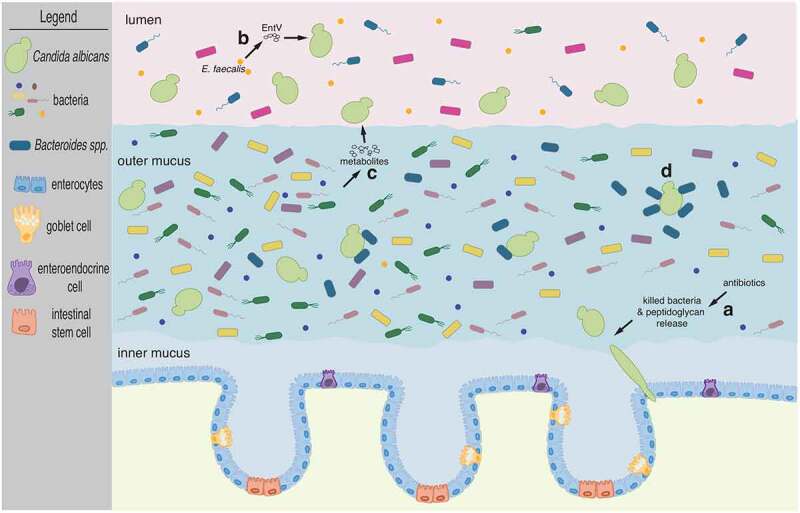
The fungus *C. albicans* inhabits the mammalian gut along with numerous and diverse bacterial species. Cartoon depicts the mammalian colon. The inner mucus layer which is largely devoid of microbes separates intestinal epithelial cells from the microbiota. *C. albicans* cells adopt the oval-shaped ‘yeast’ morphology and occupy the outer mucus layer as well as the intestinal lumen. Illustrated are four instances of documented bacterial interactions with *C. albicans*: (a) β-lactam antibiotic treatment promotes *C. albicans* filamentation due to the release of bacterial peptidoglycan subunits in the intestinal lumen. *Candida* hyphae disseminate from the gut seeding bloodstream infections. (b) The bacterium *E. faecalis* secretes the EntV peptide which inhibits hyphal morphogenesis. (c) Multiple bacterial species can provide a wide range of metabolic products which can alter *C. albicans* proliferation, albeit by unknown mechanisms. And (d) *Bacteroides* spp. closely associate with *C. albicans* cells in the outer mucus layer and can feed on mannan, a complex carbohydrate decorating the cell surface of the fungus. See main text for further details

Some of the most recognizable bacterial components are those found in their outer membrane and cell wall. Molecules derived from these structures are also among the most abundant microbial products in the gut.^44^ Peptidoglycan, for example, is a major component of the bacterial cell wall. It consists of alternating β-(1,4)-linked *N*-acetylglucosamine and *N*-acetylmuramic acid cross-linked by regularly spaced short peptides. Certain bacterial peptidoglycan subunits such as 1,6-anhydro-*N*-acetylmuramyl peptides are strong hypha-inducing agents in *C. albicans*.^45^ This is consistent with the observation that the amino sugar *N*-acetylglucosamine also promotes hyphal morphogenesis.^46,47^ In a recent study in mice,^48^ it was shown that β-lactam antibiotic treatment promoted *C. albicans* hyphal growth in the gastrointestinal tract due to the release of bacterial peptidoglycan subunits in the intestinal lumen (Figure 1a). Furthermore, the authors established that the filamenting *C. albicans* cells under these conditions could disseminate from the gut causing invasive candidiasis. The β-lactam mediated effect on the fungus could explain, at least in part, the general observation that antibiotic treatment constitutes a major risk factor for *Candida* dissemination from the gut.

Bacteria often secrete peptides and/or toxins that can act on nearby cells. *Enterococcus faecalis*, a Gram-positive, commensal bacterium inhabiting the gastrointestinal tract of humans, produces a 170-amino-acid prepropeptide which is processed by several enzymes to generate and secrete an active 68-amino-acid peptide known as EntV.^49^ The secreted peptide has been shown to inhibit the formation of *C. albicans* filaments (Figure 1b), preventing biofilm formation.^50^ While it remains to be established whether EntV has any role in the interplay between these two species in their natural habitat [*i.e*. within the mammalian intestine] this study is significant because it established the molecular basis of an antagonistic relationship between *E. faecalis* and *C. albicans* which had originally been discovered in a co-infection model.^51^

Metabolic products derived from gut bacteria may be rationalized as the most likely molecules directly affecting *C. albicans* proliferation in the intestine (Figure 1c). Indeed, metabolites produced by a consortium of 60 bacterial strains derived from human feces and grown in a continuous-culture bioreactor system inhibited to some extent the proliferation of *C. albicans* in liquid culture and also its ability to filament.^52^ The identity of these metabolites and the specific bacterial species producing them, however, remains to be established. Short-chain fatty acids (SCFAs) [in particular acetic, butyric and propionic acid] have been shown to have multiple effects on *C. albicans* under *in vitro* culture conditions: SCFAs inhibit germ tub formation, reduce metabolic activity in biofilms and impair growth.^53^ Acetic, propionic and cis-5-dodecenoic acid also reduce *C. albicans*-induced damage of cultured human epithelial cells.^16^ While metabolites produced by bacteria not typically associated with the gut, such as *Pseudomonas aeruginosa*, have been shown to directly target *C. albicans* functions,^54,55^ there is still a significant gap in our knowledge of metabolic products released by bacteria in the intestinal milieu that directly influence intestinal colonization by *C. albicans*.

While the majority of studies on bacterial–fungal interactions have looked on bacteria having effects on the fungus, it is important to notice that *C. albicans* can also have a significant influence on co-habiting bacteria. For instance, *Candida* enhances the *in vitro* growth of the strict anaerobes *Bacteroides fragilis* and *Bacteroides vulgatus*.^56^ The authors of this study observed similar effects when supplied spent *C. albicans* medium or dead *C. albicans* cells, which led them to postulate that carbohydrates located on the fungal surface could be fueling bacterial growth. Consistent with this hypothesis, *Bacteroides* spp. have been shown to harbor enzymes that are specialized in breaking down the sugar mannan,^57^ which is abundant on *Candida*’s surface. The simpler sugars resulting from the mannan digestion by the *Bacteroides* enzymes fuel the proliferation of *Bacteroides*.

The examples outlined in this section clearly indicate that gut bacteria-derived molecules, including cell wall components, peptides, and metabolic products, directly impinge upon multiple *C. albicans* traits including its proliferation in the intestine. Likewise, molecules derived from the fungus can also have a significant influence on co-habiting bacteria.

## Host-mediated effects of bacteria on *C. albicans* gut colonization

Priming responses by the host’s immune system is a well-established mechanism whereby commensal bacteria protect the mammalian host from invading pathogens.^58^ This ‘host-mediated’ or ‘indirect’ mechanism has often been invoked in the context of bacteria–bacteria antagonistic relationships. Here I briefly describe two studies that have examined the basis of antagonistic relationships between bacteria and *C. albicans* in murine models of intestinal colonization and that indicate that the host’s immune system is involved. Such evidence suggests that the concept of ‘host-mediated’ interactions can be extended to cases of fungus-bacteria interplay as well.

In one of the studies,^59^ *C. albicans* was found to protect against lethal murine *Clostridium difficile* infections. The effect appeared to be mediated, at least in part, by the fungus promoting the production of IL-17, a pro-inflammatory cytokine. In the other study,^60^ several Bacteroidetes and clostridial Firmicutes were found to antagonize *C. albicans* in the murine intestine. The authors convincingly showed that these bacteria restricted proliferation of the fungus by stimulating the production of gut mucosal immune defenses, in particular the production of the antimicrobial peptide CRAMP, which targeted *C. albicans*. It is noteworthy that, based on the two studies described here, *C. albicans* has dual roles: First, as a commensal that can elicit a response to protect the host from a bacterial pathogen; and, second, as a fungal pathogen from which the host is protected via immune responses elicited by commensal bacteria.

In addition to the two cases described in the previous paragraph, it is worth mentioning a study in a model of recurrent mucosal *Candida* infection in antibiotic-treated mice.^61^ The authors found that SCFA administration in these animals resulted in fungal clearance and inflammation resolution. Antibiotic administration dramatically reduced the intestinal regulatory T cell population (Foxp3+ T_regs_) whereas SCFA treatment partially restored its numbers. Regulatory T cells, therefore, may also be part of the bacteria-modulated host cells that shape responses to *C. albicans*.

## Gut bacteria shape the intestinal microhabitats occupied by *C. albicans*

Gut microbes inhabit a variety of distinct microhabitats along the longitudinal and cross-sectional axes of the intestinal tract.^62^ Along the intestine, mucus provides a physical barrier that separates the gut lumen from the intestinal epithelial cells.^63^ The mucus layer is a gel-like structure that represents a niche vastly different from the adjacent lumen.^64–66^ *C. albicans* has been imaged throughout the digestive tract (from stomach to small and large intestine) in both gnotobiotic and antibiotic-treated conventional mice.^67,68^ High-resolution microscopy analyses conducted with colon sections derived from gnotobiotic mice co-colonized with *C. albicans* and single bacterial species indicate that a significant proportion of fungal cells (roughly 50%) localize to the outer mucus.^69^ Fungal (in the round ‘yeast’ form) and bacterial cells (*Bacteroides thetaiotaomicron* in particular) were visualized in close association within the mucus (Figure 1d). In this experimental system, the formation of a substantial outer mucus layer was dependent on the presence of *B. thetaiotaomicron* suggesting that the spatial distribution of *C. albicans*, at least in the colon, is shaped by gut bacteria.

The partition of *C. albicans* in at least two distinct compartments, one luminal and another inside the mucus layer, raises the possibility that they represent disparate cell populations, each expressing a different set of genes. That is, the fungus could up- or down-regulate genes (to turn on or turn off associated biological functions) depending on whether it is in the lumen or mucus. In support of this notion, it has recently been shown that the gut bacteria *B. fragilis* expresses different sets of genes depending on the particular intestinal niche where the bacteria reside, namely lumen, mucus or closer to the epithelium.^70^ Cell-to-cell variability in gene expression has been observed within *C. albicans* clonal populations. In fact, the expression of a major regulator of gut colonization, the transcription regulator Efg1p, exhibits extensive cell-to-cell variability.^71^ It is tempting to speculate that such variability may, at least in part, be related to the particular microhabitat that the fungus occupies in the colon. Experimental approaches designed to carry out single-cell gene expression measurements spatially within specific gut microenvironments – such as the one described for *B. fragilis* in Ref. 70 – will allow researchers to explore these questions.

## Metabolic niche occupied by *C. albicans* in the intestine

A fundamental question regarding *C. albicans* dwelling in the gut – and more generally about all fungi that reside in this organ – is what metabolic niche the species occupies. That is, what are the primary sources of key nutrients such as carbon and nitrogen that *Candida* depends on in the gut? And, at the other end, what metabolic products does *Candida* make available to nearby microbes? Answers to these questions could provide a basic framework to infer potential interdependencies of this fungus with specific gut bacteria. The metabolic niche(s) occupied by each member of the microbiota is shaped both by nutrient availability as well as the presence of other competing or cooperating species.^72^ For example, bacteria of the genus *Bacteroides* are able to use a wide range of polysaccharides^73,74^ and a number of closely‐related species can co‐exist in the gut by cross‐feeding, which may result in complete polysaccharide utilization.^75^ While *in silico* metabolic modeling has been used to predict metabolic interactions between *C. albicans* and gut bacteria,^76^ the niche of the fungus in the mammalian gastrointestinal tract remains largely undefined. (Based on exchange reaction fluxes, the *in silico* modeling^76^ predicts, for example, that the amino acids proline and aspartate, and molecules such as nitrite and putrescine, constitute the top differences when it comes to consumption by fungus or bacteria.)

On the primary nutrient sources that *C. albicans* may use in the intestine, it has been argued that metabolic flexibility and mixed‐substrate utilization are common strategies for survival in the face of ever‐present nutrient fluctuations.^72^ Metabolic flexibility is indeed a hallmark of *C. albicans*, and has been hypothesized to contribute to survival in the mammalian host, particularly in the gut.^77,78^ Transcriptome profiling experiments that have compared *C. albicans* cells growing inside the mouse intestine to cells cultured under laboratory conditions have revealed the upregulation of genes associated with translation, sugar transport and stress responses, among others.^79,80^ These findings point to the presence of metabolically active fungal cells that take up simple sugars (monosaccharides) in the cecum. *C. albicans* is unique among studied yeasts in that it can continuously assimilate nonpreferred carbon sources even in the presence of glucose.^78,81^ The mechanism for this phenomenon involves, at least in part, the rewiring of ubiquitination targets^81^ and is predicted to make the fungus more fit in an environment in which carbon availability is constantly changing in both quantity and quality.^78^

On the metabolic products that the fungus may contribute to the intestinal milieu, a recent metabolome study comparing the gut content of antibiotic-treated mice carrying or not *C. albicans* found surprisingly few, if any, differences caused by the presence of the fungus.^82^ The ~100 metabolites screened in this study included amino acids, carbohydrates, lipids, peptides, xenobiotics and bile acids. Another study found that the presence of *C. albicans* altered the levels of several lipid species in the mouse cecum, including non-esterified, unsaturated long-chain fatty acids.^83^ Clearly, more comprehensive metabolome studies, ideally carried out in gnotobiotic animals, are needed to start delineating the metabolic footprint of the fungus in the intestine.

## Potential for translational research

*Candida albicans* and related species such as *C. parapsilosis* are opportunistic pathogens. That is, they reside in the human intestinal tract as commensals, without causing any overt disease. Occasionally, however, and typically in individuals with debilitated defenses, these *Candida* species can disseminate from the intestine and cause deep-seated, invasive infections. Because they reside in the gut, it has been hypothesized that the intestinal microbiota may play a critical role in *Candida*’s pathogenesis. Recent clinical evidence supports this notion. In patients with disseminated *Candida* infections, *C. albicans* and *C. parapsilosis* translocation into the bloodstream was found to be preceded by an expansion of both species in the gastrointestinal tract.^31^ Furthermore, fungal dysbiosis was found to be tightly associated with bacterial dysbiosis, particularly the loss of anaerobic bacteria.^31^ These observations suggest that it may be possible to devise interventions that target the gut microbiome to inhibit *Candida* dissemination from the gastrointestinal tract in people at risk of infections. Elucidating the principles that govern the interplay between gut fungi and bacteria, therefore, has the potential to open the door to a novel way of treating and preventing infections: Rationally designed, microbiota-based therapeutics.^84^ Studies on infections caused by the bacteria *Vibrio cholerae*^85^ and *Clostridium difficile*^86^ indicate that such approaches may indeed be feasible.

## Conclusions and Outlook

There is growing evidence that significant interactions take place between fungi and bacteria in the mammalian gastrointestinal tract. Yet only limited progress has been made on the actual molecular mechanisms underlying specific interactions. The enormous complexity of the mammalian gut microbiota as well as the lack of a convenient and tractable animal model of gut fungal colonization may account for the paucity of mechanistic studies dissecting physiologically relevant interactions directly in the intestine. Nevertheless, future studies exploring the interplay between *C. albicans* and other members of the microbiota are likely to reveal novel insights into inter-kingdom microbial interactions that occur in our mucosal surfaces; bacterial *vs*. fungal strategies for colonizing the mammalian gut; and, rational microbiota-targeted approaches to prevent and treat infections.

## Data Availability

Data sharing is not applicable to this article as no new data were created or analyzed in this study.
